# Feasibility of self-sampled dried blood spot and saliva samples sent by mail in a population-based study

**DOI:** 10.1186/s12885-015-1275-0

**Published:** 2015-04-11

**Authors:** Amrit Kaur Sakhi, Nasser Ezzatkhah Bastani, Merete Ellingjord-Dale, Thomas Erik Gundersen, Rune Blomhoff, Giske Ursin

**Affiliations:** 1 Norwegian Institute of Public Health, 0456 Oslo, Norway; 2 Department of Nutrition, Institute of Basic Medical Sciences, University of Oslo, 0316 Oslo, Norway; 3 Vitas AS, Oslo Innovation Park, N-0349 Oslo, Norway; 4 Department of Clinical Service, Division of Cancer Medicine, Surgery and Transplantation, Oslo University Hospital, 0424 Oslo, Norway; 5 Cancer Registry of Norway, P.O. Box 5313, 0304 Oslo, Norway; 6 Department of Preventive Medicine, University of Southern California, 90032-3628 Los Angeles, CA USA

**Keywords:** Dried blood spots, Saliva, Postal service, Carotenoids, Vitamin D

## Abstract

**Background:**

In large epidemiological studies it is often challenging to obtain biological samples. Self-sampling by study participants using dried blood spots (DBS) technique has been suggested to overcome this challenge. DBS is a type of biosampling where blood samples are obtained by a finger-prick lancet, blotted and dried on filter paper. However, the feasibility and efficacy of collecting DBS samples from study participants in large-scale epidemiological studies is not known. The aim of the present study was to test the feasibility and response rate of collecting self-sampled DBS and saliva samples in a population–based study of women above 50 years of age.

**Methods:**

We determined response proportions, number of phone calls to the study center with questions about sampling, and quality of the DBS. We recruited women through a study conducted within the Norwegian Breast Cancer Screening Program. Invitations, instructions and materials were sent to 4,597 women. The data collection took place over a 3 month period in the spring of 2009.

**Results:**

Response proportions for the collection of DBS and saliva samples were 71.0% (3,263) and 70.9% (3,258), respectively. We received 312 phone calls (7% of the 4,597 women) with questions regarding sampling. Of the 3,263 individuals that returned DBS cards, 3,038 (93.1%) had been packaged and shipped according to instructions. A total of 3,032 DBS samples were sufficient for at least one biomarker analysis (i.e. 92.9% of DBS samples received by the laboratory). 2,418 (74.1%) of the DBS cards received by the laboratory were filled with blood according to the instructions (i.e. 10 completely filled spots with up to 7 punches per spot for up to 70 separate analyses). To assess the quality of the samples, we selected and measured two biomarkers (carotenoids and vitamin D). The biomarker levels were consistent with previous reports.

**Conclusion:**

Collecting self-sampled DBS and saliva samples through the postal services provides a low cost, effective and feasible alternative in epidemiological studies.

**Electronic supplementary material:**

The online version of this article (doi:10.1186/s12885-015-1275-0) contains supplementary material, which is available to authorized users.

## Background

A common challenge for large epidemiological studies is obtaining and transporting biological samples. This challenge is especially true for blood samples. Trained personnel are required to take blood samples, and thus participants either need to visit doctor´s offices or specialized blood drawing centers, or study personnel need to visit the participants. Furthermore, blood samples typically must be shipped directly from the medical center to the receiving laboratory overnight in order to ensure the stability of the biomarkers. To overcome some of these challenges it has been suggested that participants could self-sample dried blood spots (DBS) for blood analysis and saliva samples for DNA analysis, and ship such specimens by postal service directly to the laboratory.

DBS is a form of biosampling where blood samples obtained by a finger-prick lancet are blotted on filter paper [1]. The DBS sample should be dried before being sent by regular mail, and transferred to −80°C for long term storage at the receiving laboratory. Most biomarkers are stable in DBSs for months or years at ambient or refrigerator temperatures, and for even longer periods at −80°C. The DBS platform is especially advantageous in studies of infants and small children since it is minimally invasive and small volumes often are available [2,3].

The feasibility of collecting such DBS samples from study participants in large-scale epidemiological studies is not known. Although the DBS analysis platform is routinely used for DNA, protein, virus, drugs and blood sampling in clinical practice [4-7], only a few studies have reported on the feasibility of postal collection of DBSs in population-based studies [8-10]. The expected response proportion is not known in large epidemiological studies. Specifically, it is not clear whether participants would be reluctant to take their own blood samples. It is also not known whether participants would be able to understand written instructions for obtaining and shipment of the blood sample adequately, and to what extent participants would contact study personnel with questions about the DBS protocol.

The aim of this study was to measure the feasibility of collecting self-collected DBS and saliva samples in a population-based study, where participants would be asked to ship the samples by standard postal service. Feasibility was measured by response proportion, the number of phone calls, number of adequate blood spots submitted and the quality of the blood samples.

To determine the quality of mailed DBS samples, we analyzed two key biomarkers, carotenoids and vitamin D (25-hydroxy-D3), in a subset of samples. Blood carotenoids may serve as biomarkers for fruit and vegetable intake [11-13]. They are lipid-soluble plant pigments with antioxidant activities [14]. Lutein, zeaxanthin, β-kryptoxanthin, α-carotene, β-carotene, and lycopene are among the most studied carotenoids due to their abundance in food and plasma. Vitamin D is a fat-soluble secosteroid. Sun exposure plays a central role in vitamin D metabolism, as it is formed in the skin under the influence of UV light [15-17]. Both carotenoids and vitamin D are important biomarkers in epidemiological studies of nutrients and disease.

## Methods

### Subjects and Study Design

The present study was part of a larger project on diet and breast cancer in Norway [18]. The main aims of the large project were to gain insight into the effects of women’s diet, genetics and hormones on the breast tissue, as monitored through mammographic density.

**Figure 1:**
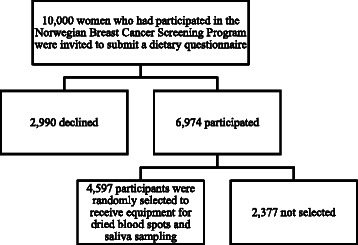
Study population overview.

In 2006 and 2007, the Norwegian Breast Cancer Screening Program included a question in their standard questionnaire sent with the invitation letter for the mammographic screening appointment on whether the woman was willing to complete a dietary questionnaire, and receive blood and saliva sampling kits. A food frequency questionnaire (FFQ) was mailed to a random sample of 10,000 women who agreed. Out of them, 6,974 returned the dietary questionnaire. Blood and saliva sample collection kits were mailed to a random sample of 4,597 of those women who had returned the questionnaire, in the spring of 2009. This study was conducted over a period of about 3 months. The inclusion and characteristics of the study participants are shown in Figure 1 and Table 1.

**Table 1:** **Characteristics of the study participants**

Overall (N = 4597)			Adequate/valid blood samples			Inadequate blood samples or did not return blood samples		
			(N = 3038/66%)^1^			(N = 1559/34%)		
**Variables**	**N**	**Mean (SD)** ^**2**^	**N**	**%**	**Mean (SD)** ^**2**^	**N**	**%**	**Mean (SD)** ^**2**^
**Age (years)**	4573	57 (4.7)	3014		57 (4.1)	1559		57 (5.1)
50-54	1499		953	32		546	35	
55-59	1528		1012	34		516	33	
60-64	1409		963	32		446	29	
65-69	137		86	3		51	3	
Chi-square p-value^**5**^	0.045							
**Body mass index (kg/m** ^**2**^ **)** ^**4**^	4243	25 (4.6)	2714		25 (4.4)	1529		25 (4.9)
<25	1562		1244	46		318	21	
>25 < 29	1680		1082	40		598	39	
>29	1001		388	14		613	40	
Chi-square p-value^**5**^	<0.001							
**Education (in years)**	4542		3013			1529		
<=10	830		512	17		318	21	
11-14	1830		1232	41		598	39	
15+	1882		1269	42		613	40	
Chi-square p-value^**5**^	0.007							
**Physical activity (less strenuous)** ^3^	3661		2414			1247		
**Hours per week**								
0	86		47	2		39	3	
1	422		275	11		147	12	
2 to 3	1620		1082	45		538	43	
4 to 5	917		605	25		312	25	
6+	616		405	17		211	17	
Chi-square p-value^**5**^	0.24							
**Physical activity (strenuous)**	3557		2353			1204		
**Hours per week**								
0	1521		986	42		535	44	
1	859		571	24		288	24	
2 to 3	929		638	27		291	24	
4 to 5	180		116	5		64	5	
6+	68		42	2		26	2	
Chi-square p-value^**5**^	0.32							
**Smoking**	3649		2397			1252		
Never	1583		1096	46		487	39	
Current	778		431	18		347	28	
Past	1288		870	36		418	33	
Chi-square p-value^5^	<0.001							

^1^Adequate/valid samples were samples returned in an aluminium bag with a desiccant pouch while invalid samples were samples without a dessicant pouch or aluminium bag.

^2^Unadjusted mean and standard deviation.

^3^Physical activity: less strenuous = walking, bicycling, working in the garden more strenuous = aerobic, running, bicycling at high intensity.

^4^excluded women with height <125, and weight < 30 kg >170 kg.

^5^Compared the adequate (n = 3038) with the inadequate blood samples (n = 1559).

The blood sampling kit consisted of two blood DBS cards (Protein Saver^TM^ 903^R^ Cards, Whatman, Sanford, USA), two lancets, one 5-mg desiccant pouch (Reàl Marine A/S Stavanger, Norway), one aluminum zip-lock bag (Whatman, Sanford, USA), Cutisoft® wipes, Mesoft swabs (Mölnlycke Healthcare) and one small bandage. The airtight aluminum bag was used to protect the blood sample during shipment. The desiccant bag was included to remove any moisture from the DBS cards. To suppress the degradation of carotenoids in the DBS samples [1], the first two circles in the DBS cards were impregnated with a proprietary stabilizing solution supplied by Vitas AS, Oslo, Norway. The saliva sampling kit consisted of a saliva collection tube and a bag, Oragene™ DNA Self-Collection Kit (DNA Genotek Inc., Kanata, ON, Canada). The bag protected the saliva sample during mailing. Detailed instructions for blood and saliva sample collection were mailed together with the sample collection kits (Additional file 1).

### Blood Samples

#### Validity of blood samples

Upon receipt, the DBS cards were assessed by a trained research assistant for both validity and amount of blood in each spot. Samples were considered valid if and only if they were shipped in aluminum bags with the desiccant pouch. The amount of blood received was assessed by the number and size of the blood spots. Samples were classified into three categories: (a) filled, (b) small and (c) empty blood spots (a spot is the area within the circle, 13 mm in diameter that is supposed to be filled with blood). In a “filled blood spot” the spot was completely or almost completely filled with blood. Such a spot contained approximately 50 μl of blood and was enough for about 7 punches. A punch is 3.2 mm in diameter and would provide 3.1 μl of blood [19]. A “small blood spot” was defined as a blood spot sufficient for only one punch. An “empty blood spot” was defined as a blood spot with less blood than 3.2 mm in diameter or completely empty. The DBS cards with blood were stored in the laboratory at −80°C.

#### Analysis of blood samples

A subset of 381 valid samples was selected for analysis of vitamin D and carotenoids (lutein, zeaxanthin, β-kryptoxanthin, α-carotene, β-carotene and lycopene). The 381 samples were selected based on the following inclusion criteria: age at screening >50 years, energy intake >2100 kJ and <15000 kJ and body mass index > 15 kg/m^2^ and <50 kg/m^2^.

High-performance liquid chromatography with ultraviolet detection and liquid chromatography-mass spectrometry were used for analysis of carotenoids and vitamin D, respectively [20,21].

Hematocrit values in normal adult women are about 50%. In order to compare DBS results with results from analysis of plasma, all DBS values were multiplied with a factor of 2 [2].

#### Statistics

We used excel to calculate unadjusted chisquare tests for the overall differences in proportions (test for homogeneity). All tests of significance were 2-sided and p < 0.05 was considered statistically significant. The IBM Statistical Package for Social Sciences (SPSS) was used for calculating frequencies in Table 2 [Version 20 (IBM Corp 2012) Armonk, NY:IBM Corp].

**Table 2:** **Number of participants submitting adequately filled spots and blood spots allowing at least one punch for analysis**

Number of blood spots	Number of participants with adequately filled blood spots	Number of participants with blood spots allowing at least one punch^1^
10	2,418	2,655
≥9	2,521	2,729
≥8	2,613	2,796
≥7	2,692	2,850
≥6	2,750	2,896
≥5	2,834	2,964
≥4	2,871	2,988
≥3	2,906	3,011
≥2	2,938	3,026
≥1	2,960	3,032

^1^a punch is 3.2 mm in diameter and would provide 3.1 μl of blood.

#### Ethics statement

The present study was conducted according to the Declaration of Helsinki guidelines and approved by The Regional Committee for Medical Research Ethics. All the participants gave their written informed consent.

## Results

**Figure 2:**
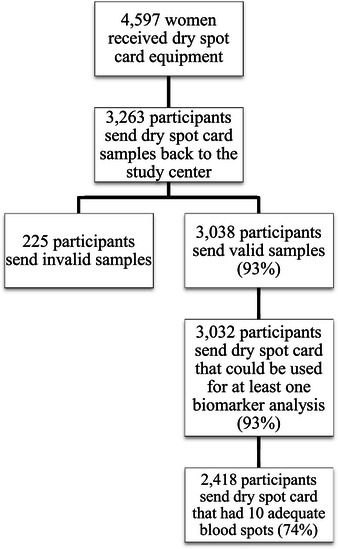
Response proportions in study where DBS cards were shipped to 4597 women who had returned a dietary questionnaire.

Of the 4,597 sampling kits sent to participants, we received DBS samples from 3,263 women (71.0%) (Figure 2) and saliva samples from 3,258 women (70.9%). A total of 117 (2.5%) of the 4,597 mailed kits were returned due to erroneous addresses and 12 were lost during the mailing process.

**Figure 3:**
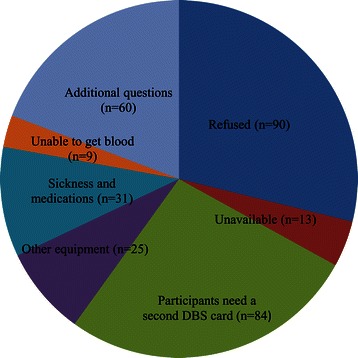
Phone calls from 312 out of the 4,597 participants.

We received 312 (6.8%) phone calls from the 4,597 participants. Reasons for the phone calls included the following: participants that refused (n = 90) or were unavailable to participate of other reasons (n = 13), participants needing a second DBS card (n = 84) or other equipment (n = 25), sickness and medications (n = 31), participants not able to get blood after finger-prick (n = 9) and additional questions or reasons (n = 60) (Figure 3).

**Figure 4:**
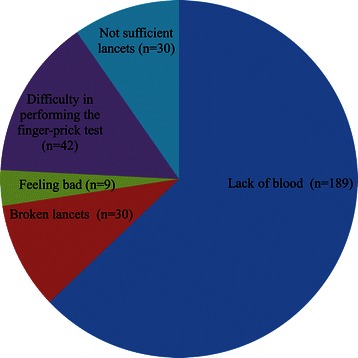
Written comments from 300 out of 3,263 participants.

Of the 3,263 women who submitted the DBS samples, a total of 300 participants (9.2%) wrote comments on the form included with the sampling kit (Figure 4). Most of these comments were regarding lack of blood (n = 189) and difficulty in performing the finger-prick test (n = 42). Some comments were also about broken lancets (n = 30), insufficient number of lancets (n = 30). Only a small number reported feeling unwell when performing the finger-prick test (n = 9).

Out of 3,263 received DBS samples, 3038 (93.1%) were packed and shipped as instructed, while 225 participants (Figure 2) either omitted the desiccant pouches or failed to place the DBS cards in the aluminum bags as instructed. Because this could affect the stability of the biomarkers, we classified these as inadequate or invalid blood samples. There were a number of differences between the 3038 women with adequate samples and the 1559 women who did not return a sample or who returned an inadequate sample (Table 1). Those with valid samples tended to be slimmer, more highly educated and less likely to be current smokers.

Additionally, a few participants (n = 6) submitted DBS cards that only contained empty spots or spots with less blood than required for a single punch. Out of 3,263 DBS cards submitted to the laboratory, 3,032 (92.9%) could be used for at least one biomarker analysis (Table 2). Table 2 also presents the number of participants that were able to submit DBS cards with 1–10 adequately filled blood spots (with each spot enabling up to 7 punches for individual analysis) and 1–10 blood spots which allow at least one punch for analysis. Thus, 2,418 (74.1%) DBS cards were returned with all 10 spots filled with blood according to the instructions. These DBS cards will allow up to 70 punches for separate analyses from each participant.

Measurements of carotenoids and vitamin D in a subset of 381 samples are shown in Table 3, where we also list results obtained in previous studies from Norway or Nordic countries [20,22-25]. The results demonstrate that plasma values are similar to those obtained in previous studies. One exception was lycopene, which was somewhat lower in this study than in the other studies, but higher than in the study from Finland.

**Table 3:** **The mean concentration of carotenoids and 25-hydroxy vitamin D**_**3**_ **from DBS samples compared with plasma from other studies in theNordic countries**

	Lutein*(μmol/L)*	Zeaxanthin*(μmol/L)*	β-kryptoxanthin*(μmol/L)*	α-carotene*(μmol/L)*	β-carotene*(μmol/L)*	Lycopene*(μmol/L)*	25-hydroxy D3*(nmol/L)*	N (Carotenoids - vitamin D=	References (Carotenoids – vitamin D)
**Norway – DBS samples present study mean (SD)** ^**1**^	0.23 (±0.13)	0.046 (±0.02)	0.16 (±0.11)	0.13 (±0.10)	0.43 (±0.29)	0.25 (±0.12)	43 (±12)	403 - 403	The present study
**Norway previous study mean (SD)**	0.17 (±0.07)	0.04 (±0.02)	0.16 (±0.11)	0.14 (±0.12)	0.50 (±0.32)	0.63 (±0.33)	n.a	346 – n.a.	[20]
**Denmark study mean (SD)**	0.34 (±0.14)	0.07 (±0.04)	0.23 (±0.21)	0.22 (±0.18)	0.47 (±0.38)	0.53 (±0.29)	75 (±29)	98 – 2,016	[22,23]
**Sweden study mean (SD)**	0.28 (±0.12)	0.06 (±0.04)	0.20 (±0.19)	0.20 (±0.22)	0.54 (±0.73)	0.52 (±0.27)	69 (±23)	97 – 116	[22,23]
**Finland study mean (SD)**	0.20 (±0.10)	0.04 (±0,02)	0.20 (±0.18)	0.19 (±0.13)	0.69 (±0.47)	0.09 (±0.06)	38.1 (±4.6)	620 – 1,283	[25,28]

Values are means and SD (standard derivation).

n.a. = not analyzed.

^1^In order to compare DBS results with results from analysis of plasma, all DBS values were multiplied with a factor of 2.

## Discussion

In the present study, we found that by sending out DBS and saliva sample collection kits with instructions to women aged 50–69 who had agreed to participate in a dietary study, self-collected samples were received from about 70% of the participants. The collection resulted in phone calls from about 7% of women, where about a third was related to the lancets, or difficulties in using them. Of the blood samples received, about 93% were considered valid and could be used for at least one biomarker analysis. Overall 74% had 10 filled spots that would we used for up to 70 separate blood analyses. Measurement of two selected biomarkers showed similar results to those obtained in other studies.

The participation rate in this study of self-sampling was good. The fact that response proportion was similar for the DBS samples and saliva samples, suggests that those who are willing to provide a biological sample are also willing to do so, even if it entails a finger prick. However, women who provided DBS and saliva samples had agreed to participate in the study and had also completed a dietary questionnaire. One could argue that the relatively high proportion that responded represented a highly motivated group. Further, women with an adequate/valid sample were more highly educated and healthier than those who did not participate or had an invalid sample. The largest difference was found for smoking, confirming the participants represented a selected group. A case–control study from the US that included a $ 2.00 bill to encourage participation, yielded similar participation (68%), and found that the participation with DBS was better than venipuncture (62%) [8]. Their study was, however smaller, with 134 female cancer cases and 256 controls. In the present study we did not include a cash incentive, but still obtained a participation rate of 70% among those who had already returned a dietary questionnaire.

We also determined the usefulness or quality of DBS cards returned to the laboratory. Based on our assessments, about 93% of the received DBS cards had sufficient blood spots for at least one biomarker analysis and most of these had 10 adequate spots.

Only about 7% of the participants contacted the study center by phone. Although a third of these were refusals, about a third were regarding lack of or malfunctioning equipment (lancets). The study staff tested out a series of lancets in advance, both internally and in a pilot, before deciding on the one that was the most reliable. Since several participants still complained about the lancet, any future study should test in more detail several lancets before commencing a large epidemiological study, or consider including two lancets.

The levels of the biomarkers (vitamin D and carotenoids) analyzed in this study were compared with findings from other studies to confirm the reliability of DBS to plasma analysis of biomarkers. The concentrations of these biomarkers in human blood vary across Europe [22,23]. We compared our results with those in similar population samples (women, comparable age) from studies in Nordic countries [23-28]. Levels of all biomarkers analyzed in the present study, were similar with those from other studies. The levels of lycopene in the present study were somewhat lower than three other studies but higher than a study from Finland. These variations probably reflect different dietary intake of tomato products like tomato sauce, pizza and ketchup [29] in the different populations, since these foods are the major sources for lycopene.

Some caution must be taken when comparing DBS data with plasma analyses performed in blood samples taken by venipuncture. Absolute values from DBS samples (i.e. whole blood) are expected to be about 50% of values reported in plasma [2], because whole blood includes blood cells as well as plasma. In normal adult women, hematocrit values are about 50%, and thus about half of the blood volume represents blood cells. Thus, in the comparison between DBS and plasma analysis, all DBS values were multiplied with a factor of 2. In addition, the recovery or extraction of biomarkers from DBS might also differ in comparison to plasma. Thus, development of separate reference ranges of different biomarkers in DBS cards is needed.

Unlike venipuncture, trained personnel were not required for DBS collection and the transportation and storage of samples was easier. The reduced storage space requirements are also a major advantage, especially when thousands of samples are to be collected in large epidemiological studies. The volume needed for storage of DBS samples is typically less than one tenth of similar aliquots of plasma samples. Furthermore, obtaining small samples for analysis is often also much simpler from DBS cards, since no thawing and refreezing of plasma samples are needed.

A major advantage of the DBS cards is reduced cost, a typical limiting factor when performing epidemiological studies. A direct comparison between the cost when using self-sampled DBS cards and plasma samples from venipuncture by health personnel is difficult, but will in most instances be very large (e.g. reduced costs for transportation of participants to study or blood collection center, for equipment, storage and personnel).

There are a number of advantages of sample collection by postal service. It may increase participation rate in a population-based study requiring blood samples. In particular, this sample collection technique increases the possibility of obtaining samples from people living in remote rural areas. Further, the rapidness of the data collection, collecting samples from over 3000 women in less than 3 months, is a strong advantage. There are also some limitations with self-sampled DBS collection via the postal service. One of the disadvantages is that the participants must follow the instructions carefully and failure to do so may affect the results. In our study, we observed that 7% did not return the samples packaged as we had instructed with the desiccant and inside the aluminum bag.

## Conclusion

We explored the feasibility of self-sampled DBS cards and saliva samples shipped by postal service. Response proportions were 70.9% and 71.0%, respectively. Of the DBS samples obtained, over 90% were considered valid and sufficient for at least one biomarker analysis. The data collection resulted in a limited number of phone calls to the study center. Our study suggests that the DBS collection method is efficient, yields a high response proportion and blood spots that can be used in large population-based studies. Overall self-sampled DBS and saliva samples shipped through the postal service appears to offer a low cost, effective and feasible means for collecting biological samples in epidemiological studies.

## Additional file

Additional file 1:
**Dried blood sample (DBS) collection instructions.**

## Abbreviations

DBS: Dried blood spot
DNA: Deoxyribonucleic acid
