# Supplementary material for: Feasibility of self-sampled dried blood spot and saliva samples sent by mail in a population-based study
Source: BMC Cancer. 2015 Apr 11;15:265. doi: 10.1186/s12885-015-1275-0 (PMC4428002; doi:10.1186/s12885-015-1275-0)
Supplement: Additional file 1: — Dried blood sample (DBS) collection instructions. [file 12885_2015_1275_MOESM1_ESM.docx]

# Dried blood sample (DBS) collection instructions

Please read all instructions carefully before start. These instructions were prepared by Vitas AS (http://www.vitas.no/)

Take the blood test in the morning fasting that is before you have anything to eat or drink. For example, if you are going to take the blood test at 08 am, you cannot eat or drink anything after 10 pm the night before.

1. Preparation

Wash your hands in warm water with soap and dry them. Empty the envelope. Find both DBS sampling cards in the silver pouch.

**

The pouch also contains a packet (DESICCANT), please do not remove it. Close the pouch and save it for later. Put both DBS sampling cards on a clean dry surface.

2. Wipe the fingertip

Rub hands together for 10 to 20 seconds to increase blood flow. You can also swing your arm in circles a few times to increase blood flow to the hand. Open the package with the alcohol pad and wipe the selected fingertip with the alcohol pad (Figure 2).

**

3. Prick finger

Locate the lancet (blue and white plastic tool) and remove the protective blue tip. Use the lancet to prick the side of your fingertip (Figure 3).

**

4. Wipe first drop

Gently apply pressure near the puncture site to form a drop of blood. Wipe away the first drop of blood with the cotton pad (Figure 4). Let a new drop of blood form.

**

5. Apply blood drops to cards

Let the next drops fall on the circles marked on the card, one drop in each circle (Figure 5).

**

Repeat until you have filled in all the circles in both cards.

6. Fill card

**

Let the cards dry for 4 to 8 hours (Figure 6). Do not let the cards dry in direct sunlight. The circles should not be touched during drying.

7. Pack cards

When the cards have finished drying, close the card and write down the date. Put both cards in the silver pouch with the DESICCANT package. Close the pouch and place it in the return envelope along with the signed agreement and the plastic bag with the saliva sample.

**

8 Send by mail

Post the envelope the next day at the latest.

If you had any problems obtaining the blood sample, please comment here:

___________________________________________________________________________

___________________________________________________________________________

___________________________________________________________________________
